# The Importance of Differentiating Oligoarticular Juvenile Idiopathic Arthritis From Lyme Arthritis in Pediatric Patients

**DOI:** 10.7759/cureus.32785

**Published:** 2022-12-21

**Authors:** Wajiha Jeelani, Rana Harhay, Brian H Wrotniak, Thomas Hargest, Amanda Teo, Rabheh Abdul-Aziz

**Affiliations:** 1 Pediatrics, University at Buffalo, John R. Oishei Children's Hospital, Buffalo, USA; 2 Pediatric Emergency Medicine, The University of Tennessee Health Science Center, Memphis, USA; 3 Biological Sciences, University at Buffalo, Buffalo, USA; 4 Pediatric Rheumatology, University at Buffalo, John R. Oishei Children's Hospital, Buffalo, USA

**Keywords:** arthritis, jia, oligoarticular juvenile idiopathic arthritis, lyme disease, lyme arthritis

## Abstract

Objective

This study aims to compare clinical and laboratory features between Lyme arthritis (LA) and oligoarticular juvenile idiopathic arthritis (oligoarticular JIA) by examining several potential predictors in pediatric patients. This study also aims to improve and increase awareness of ways to detect LA and oligoarticular JIA in pediatric patients who present with clinical features of joint pain.

Methods

A medical chart review was conducted among pediatric patients diagnosed with LA or oligoarticular JIA at John R. Oishei Children’s Hospital of Buffalo between January 2014 and September 2018. Patients’ diagnoses were identified using the International Classification of Disease 10th Revision code for LA (ICD 10 code A69.23) and oligoarticular JIA (ICD 10 code M08.40).

Patients with LA were only included in this study if they (1) exhibited arthritis, (2) tested positive for Lyme antibodies, (3) indicated a positive western blot (WB) of five or more out of 10 *Borrelia burgdorferi* proteins by IgG antibodies or at least two of three *B. burgdorferi* proteins by IgM antibodies, and (4) at the age of 16 or below at the time of diagnosis. Patients with oligoarticular JIA were included in this study if they (1) exhibited arthritis affecting one to four joints for at least six weeks in the first six months of diagnosis and (2) are at the age of 16 or below at the time of diagnosis after ruling out LA and reactive arthritis.

In this study, clinical presentations, physical exam findings during initial healthcare visits, and demographics including age, sex, and race of patients were obtained. In addition, laboratory results including white blood cells (WBCs), hemoglobin (Hgb), platelet count, erythrocyte sedimentation rate (ESR), C-reactive protein (CRP) level, Lyme antibodies through enzyme-linked immunosorbent assay (ELISA) and WB, synovial fluid analysis for red blood cells (RBCs), nucleated cells, and polymerase chain reaction (PCR) for *B. burgdorferi* DNA were also collected and analyzed.

Results

In our data, ESR and CRP were significantly higher in LA compared to oligoarticular JIA (P=0.0053 and 0.0005, respectively). The mean WBC in the synovial joint fluid was significantly higher in LA compared to oligoarticular JIA (P=0.002).

Conclusion

LA shares features with oligoarticular JIA. This overlap prevents the creation of a clinically useful predictive model for LA. Therefore, Lyme testing should be performed on all patients presenting with monoarticular and oligoarticular arthritis. In addition, ESR, CRP, and WBC in the synovial joint fluid were significantly higher in LA compared to oligoarticular JIA in our findings. While this difference is not definitive by any means, it may help distinguish between the two in cases where the diagnosis is not clear-cut, and the values of ESR, CRP, and WBC in the joint aspirate may help guide clinical judgment in cases that lack a definitive diagnosis.

## Introduction

Lyme disease results from the infection of the spirochete *Borrelia burgdorferi* through the transmission of tick bites [1,2]. An estimated 476,000 patients are diagnosed with Lyme disease every year in the United States, with a significantly higher incidence in endemic regions in the northeast [3]. The clinical presentation of Lyme disease is heterogeneous [2]. Early stages are characterized by localized skin manifestations (erythema migrans) and a disseminated stage with multiple erythema migrans and evidence of multi-system involvement like cranial nerve palsy, meningitis, carditis, and significant systemic symptoms [4,5]. Lyme arthritis (LA) is a late manifestation of Lyme disease, which occurs in 50-60% of untreated patients [2]. LA typically presents as mono- or oligoarticular arthritis involving large weight-bearing joints, most commonly at the knee joints, often without evidence of systemic signs and symptoms [4]. The clinical presentation is similar to other inflammatory or infectious arthritis, such as septic arthritis, transient synovitis, oligoarticular juvenile idiopathic arthritis (oligoarticular JIA), or reactive arthritis [6].

In children, LA is often misdiagnosed as the oligoarticular subtype of juvenile idiopathic arthritis (JIA) [6]. JIA is the most common rheumatological disease diagnosed in children. It has an incidence of 13.9 cases per 100,000 and is most common in four to five years of age [7]. The oligoarticular subtype, also known as oligoarticular JIA, typically involves one to four joints with symptoms lasting at least six weeks [8]. Similar to LA, weight-bearing joints in the lower extremities are most frequently involved [7]. As a result, distinguishing between oligoarticular JIA and LA can be challenging. This is critical, as the treatment approaches for both diseases are vastly different.

Due to the high incidence of Lyme disease in the Western New York (WNY) region, we instituted a mandatory Lyme disease screening protocol for all patients under the age of 16 years and presenting with oligoarticular JIA symptoms in 2016, and immediately noticed a significant increase in the incidence of LA [5]. In this paper, we compare the clinical presentation of pediatric patients diagnosed with LA and oligoarticular JIA, focusing on the pattern of joint involvement, results of laboratory testing, and joint fluid analysis.

## Materials and methods

In this study, medical records of pediatric patients diagnosed with LA or oligoarticular JIA at John R. Oishei Children’s Hospital (Buffalo, New York) between January 2014 and September 2018, were retrospectively reviewed. Approval was obtained from the State University of New York (SUNY) and the University at Buffalo Institutional Review Board (UBIRB) (reference numbers: STUDY00002006 and STUDY00003014).

Patients’ diagnoses were identified using the International Classification of Disease 10th Revision Code for LA (ICD 10 code A69.23) and oligoarticular JIA (ICD 10 code M08.40).

Patients with LA were included in this study if they exhibited arthritis, tested positive for Lyme antibodies, indicated a positive western blot (WB) of at least five of 10 *B. burgdorferi* proteins by IgG antibodies or at least two of three *B. burgdorferi* proteins by IgM antibodies, and at the age of 16 or below at the time of diagnosis. Patients with oligoarticular JIA were included in this study if they exhibited arthritis affecting one to four joints in the first six months of diagnosis, at the age of 16 or below at the time of diagnosis, and had arthritis that lasted more than six weeks. 

Clinical presentations, physical exam findings during initial healthcare visits, and demographics including age, sex, and race of patients in this study were reviewed. In addition, laboratory results including white blood cell count (WBC), hemoglobin (Hgb), platelet count, erythrocyte sedimentation rate (ESR), C-reactive protein (CRP) level, Lyme antibodies through enzyme-linked immunosorbent assay (ELISA) and WB, synovial fluid analysis for nucleated cells, and polymerase chain reaction (PCR) for *B. burgdorferi* DNA were collected and analyzed. 

Descriptive statistics were computed to characterize the study sample. Categorical variables (e.g., gender, race, and joints involved) were reported as proportions in percentage, and continuous-level variables (e.g., age) as medians and interquartile ranges. Separate Mann-Whitney U tests were used to assess differences between patients with Lyme arthritis and oligoarticular juvenile idiopathic arthritis for continuous-level variables. Separate chi-square tests were used to assess differences for categorical variables between the two diseases, with Fisher’s exact test used for contingency tables having any cell less than five observations. All statistical tests were conducted as two-tailed, based on an alpha of 0.05, and with SYSTAT 13 (SYSTAT Software, Inpixon, Palo Alto, CA, 2004).

## Results

Baseline demographics

Among the 61 patients included in this study, 44 patients were diagnosed with oligoarticular JIA, and 17 patients were diagnosed with LA (Table 1). The median age of patients with oligoarticular JIA was six years (interquartile range, IQR 5.5), while the median age of patients with LA was nine years (IQR 5.5), and the difference was statistically significant (p=0.041). A large proportion of patients with oligoarticular JIA were females (70.4%) compared to patients with LA (53%), but this difference did not achieve statistical significance (p=0.197). A majority of patients in our cohort were white (82%), with similar proportions in both the oligoarticular JIA (82%) and LA (82%) groups (Table 1).

**Table 1:** Baseline characteristics. JIA: juvenile idiopathic arthritis, IQR: interquartile range.

Characteristics	All patients	Oligo-JIA	Lyme disease	p-value
Age in years, median (IQR)	7.0 (6.0)	6.0 (5.5)	9.0 (5.5)	0.041
Female, sex N (%)	40 (65.6)	31 (70.4)	9 (53)	0.197
Race, N (%)				0.654
Asian	1 (1.6)	1 (2.3)	0 (0)	
Black or African American	4 (6.6)	3 (6.8)	1 (5.9)	
Hispanic or Latino	2 (3.3)	1 (2.3)	1 (5.9)	
Mixed	2 (3.3)	2 (4.5)	0 (0)	
Native American	1 (1.6)	0 (0)	1 (5.9)	
Unknown	1 (1.6)	1 (2.3)	0 (0)	
White	50 (82)	36 (82)	14 (82)	
Clinical presentation				
Number of joints involved, N (%)				0.421
Mono-articular	44 (72.1)	33 (75.0)	11 (64.7)	
Oligo-articular	17 (27.9)	11 (25.0)	6 (35.3)	
Joints involved, N (%)				
Knee	42 (63.6)	28 (87.5)	14 (82.4)	
Ankle	7 (10.6)	4 (12.5)	3 (17.7)	
Wrist	7 (10.6)	4 (12.5)	3 (17.7)	
Elbow	2 (3.0)	2 (6.3)	0 (0)	
Hip	3 (4.5)	2 (6.3)	1 (5.9)	
Other	5 (7.6)	1 (3.1)	4 (23.5)	
Laboratory features, median (IQR)				
White blood cell count, ×10^3^/μL	8.20 (3.43)	8.20 (2.93)	8.90 (4.20)	0.3
Hemoglobin, g/dL	12.23 (9.90-14.60)	12.40 (1.70)	12.0 (1.78)	0.297
Platelets, ×10^3^/μL	339.0 (141.3)	329.0 (165.8)	346.5 (79.5)	0.985

Clinical presentation

Overall, a majority of patients (44/61, 72.1%) presented with involvement of a single joint (Table 1). There was no statistically significant difference in the number of joints affected between the two groups. In the JIA group, 75% (33/44) of patients presented with monoarticular arthritis, compared to 64.7% (11/17) of patients with LA (p=0.421). The remaining 11 patients with JIA and five patients with LA presented with oligoarticular arthritis (involvement of ≤4 joints). There is one patient with LA who presented with joint involvement in more than four joints. The time from starting symptoms of arthritis until evaluation by a rheumatologist was varied for LA from 1 to 365 days and for oligoarticular JIA was 14 days to 96 months.

The knee was the most common and affected joint in both oligoarticular JIA (87.5%) and LA (82.4%). Patients presented with only knee joint involvement in 65.6% of patients with oligoarticular JIA and 59.1% of patients with LA. The next most commonly affected joints were the ankle (10.6%) and wrist (10.6%). In an uncommon presentation for LA, arthritis was present bilaterally at the interphalangeal joint in two patients (11.7%) (Table 1).

A majority (77.3%) of patients in the cohort, arthritis was the only clinical presentation at the time of diagnosis. Within the LA group, 59.1% of patients did not have documentation or recollection of any symptoms during the early stages of Lyme disease, including no rashes, no history of a tick bite, and no flu-like symptoms during the summer, and arthritis was the initial presenting symptom. In addition to arthritis, three (13.6%) patients with LA had neurologic symptoms, including unilateral seventh cranial nerve palsy (n=1), numbness in the lower extremities (n=1), and headaches (n=1). Patients with neurological symptoms showed no symptoms of nuchal rigidity or photophobia to warranty lumber puncture. Moreover, 3 (13.6%) patients also reported the presence of a rash, but they did not seek medical care, and another 3 (13.6%) patients reported a previous history of a rash or bug bite three to four months before the presentation that had resolved. In addition, 4 (18.2%) patients reported a history of camping or residing in an area at high risk for Lyme disease, such as a wooded area. 

The median WBC count was 8.2 × 10^3^/μL (IQR 2.93 × 10^3^/μL) in the oligoarticular JIA group, compared to 8.9 × 10^3^/μL (IQR 4.20 × 10^3^/μL) in the LA group, p=0.3 (Table 1). Similarly, there were no differences in the Hb level and platelet counts between the two groups (p=0.297 and p=0.985, respectively) (Table 1). All our patients have normal transaminases. 

Inflammatory markers and joint fluid analysis

We compared levels of ESR, CRP, rates of antinuclear antibody (ANA) positivity in serum, and WBC counts in synovial fluid. A significantly higher proportion of patients with LA had elevated ESR levels compared to patients with JIA (81.3% versus 40.0%, p=0.0053). Similarly, a higher frequency of patients with LA had an elevated CRP level compared to patients with JIA (73.3% versus 21.6%, p=0.0005). We compared the median ESR and CRP levels in patients with LA and oligoarticular JIA who had elevated levels of these inflammatory markers. While the median ESR was numerically higher in patients with LA (59 mm/h) compared to oligoarticular JIA (44.5 mm/h), this did not achieve statistical significance, p=0.051. Similar results were noted in patients with elevated CRP levels. There was no significant difference in the proportion of patients with a positive ANA between the two groups (55.6% in the LA group versus 48.7% in the oligoarticular JIA group, p=0.709) (Table 2).

**Table 2:** Inflammatory markers and synovial fluid analysis. JIA: juvenile idiopathic arthritis, ESR: erythrocyte sedimentation rate, IQR: interquartile range, CRP: C-reactive protein, ANA: antinuclear antibody. *Upper normal ESR is 12 for age 1-10-year-old and 20 for age 11-18-year-old. **ANA is considered positive if the titer is 1/80 or higher.

Characteristics	All patients	Oligo-JIA	Lyme disease	p-value
ESR				
Median (IQ), mm/h	49.0 (31.8)	44.5 (28.0)	59.0 (31.3)	0.051
Elevated ESR*, N (%)	29 (47.5)	16 (40.0)	13 (81.3)	0.0053
CRP				
Median (IQR), mg/L	34.7 (30.7)	34.7 (17.3)	41.0 (65.2)	0.62
Elevated CRP, N (%)	19 (31.1)	8 (21.6)	11 (73.3)	0.0005
ANA Positive**, N (%)	24 (39.3)	19 (48.7)	5 (55.6)	0.709
Synovial fluid analysis, median (IQR)	N=17	N=10	N=7	
Mean white blood cell count, cells/μL	7530.0 (25,907.5)	4795 (5333.0)	35,550 (31,400.3)	0.002
White blood cells >10,000/μL, N (%)	8 (47.1)	2 (25)	6 (75)	0.157
White blood cells ≤10,000/μL, N (%)	9 (52.9)	8 (88.9)	1 (11.1)	0.02

Joint fluid analysis was available for 10 out of 44 patients in the oligoarticular JIA group and seven of 17 patients with LA. The median WBC count in synovial fluid was significantly higher in the LA group compared to the oligoarticular JIA group (35,550 cells/uL versus 4795 cells/uL, p=0.002). The synovial fluid WBC count was >10,000/uL in eight patients (two with oligoarticular JIA and six with LA, p=0.157). However, a higher proportion of patients with synovial fluid WBC <10,000/uL had oligoarticular JIA (eight with oligoarticular JIA and one with LA, p=0.02) (Table 2).

## Discussion

In this study, we describe the clinical and laboratory features of a series of pediatric patients presenting to the John R. Oishei Children’s Hospital with a diagnosis of LA and oligoarticular JIA. Despite the different etiologies, we found considerable overlap between the clinical presentation of these two diagnostic entities, with no difference in joint involvement and the absence of a typical history in many of our patients with LA. Importantly, we highlight that despite these similarities in clinical presentation, patients with LA have significant elevations in inflammatory markers and synovial fluid leukocytosis, features that should lead to greater suspicion of this entity. Lastly, our study highlights the importance of Lyme ELISA testing in pediatric patients who present with mono- or oligoarticular arthritis even in the absence of a history of tick bites or localized Lyme disease, given the significant increase in the number of Lyme disease cases in Western New York [5]. 

LA can occur at any age, with the average age of children being 8.59 years old [6,9,10]. Previous studies on Lyme disease and LA have also shown a higher incidence in males, which is in line with our findings. In contrast, oligoarticular JIA appears to have a strong predilection for females under the age of four [11]. While the difference in the median age at presentation was significant, there was no difference in sex between patients with LA and oligoarticular JIA in our cases.

We found a significant overlap in the clinical presentation of patients with LA and oligoarticular JIA, with the predominant involvement of a few large joints, most commonly the knee joint. There was no difference in the number or pattern of joint involvements between the two disease entities. This is again consistent with the findings of other groups reported in the literature, where large joints are predominantly involved in patients with both oligoarticular JIA and LA [6,11]. In oligoarticular JIA, the knee joint is commonly involved, followed by the ankle joint [11,12]. Other joints, such as fingers, wrists, and the cervical spine, are rarely involved in oligoarticular JIA. Patients with LA typically have less than two joint involvement, with the knee being the most commonly affected joint [6,9,13]. Pain in the joints is often minimal in both diseases, despite significant swelling. The majority of the patients in both LA and oligoarticular JIA were typically affected by only a single joint.

Importantly, we found that 59.1% of patients with LA had arthritis as their first and only clinical presentation, with no evidence of previous localized Lyme disease or recollection of other important risk factors for Lyme disease. These findings are consistent with those reported in the literature, where arthritis was the presenting symptom in approximately 60% of untreated Lyme disease cases, and highlight the importance of maintaining a high suspicion for this diagnosis in endemic regions [10,14].

We compared laboratory features, including ANA and inflammatory markers, in both groups. Studies have shown that ANAs can be positive in up to 70% of oligoarticular JIA patients [15]. In our study, a positive ANA did not help distinguish between oligoarticular JIA and LA. While data were not available for all patients, we found that 19 patients, 48.7% of patients with oligoarticular JIA had a positive ANA, compared to five patients, 55.6% of patients with LA.

Elevations in inflammatory markers (ESR and CRP) were more frequently noted in patients with LA (81.3% and 73.3%, respectively) compared to oligoarticular JIA (40.0% and 21.6%, respectively). However, the IQR varied considerably among patients. In addition, not all patients with LA had elevated inflammatory markers, and similarly, significant ESR and CRP elevations were seen in some patients with oligoarticular JIA. These findings are consistent with the reported literature, where ESR and CRP elevations are often but not always seen in patients with LA, highlighting the importance of not excluding this diagnosis in the presence of normal ESR and CRP levels [6,10,16]. The mean ESR levels of LA patients reported in the literature typically range from 39 to 44.6, and the mean CRP levels range from 2.7 to 38.5 [6,10,16]. Patients with oligoarticular JIA frequently have an elevated ESR and CRP, typically with averages around 28-34 and 9.1, respectively [12]. This highlights the importance of Lyme antibody testing and WB analysis to make a definite diagnosis of one over the other and should therefore be performed in every suspected case of LA, particularly in regions with a high incidence of Lyme disease.

We also noted that patients with LA had a higher median synovial fluid WBC count and more frequent elevations in the synovial fluid WBC count compared to patients with oligoarticular JIA, another potentially important marker to suggest the diagnosis of LA. There are other studies in the literature comparing synovial fluid markers in Lyme disease versus septic arthritis or Lyme disease among pediatric and adult populations, but none with such a direct comparison between Lyme disease and oligoarticular JIA [6,10].

Our study has a few limitations. Firstly, our sample size is small. Second, our data are based on a retrospective chart review at a single institution. Despite this, our study highlights the significant overlap in clinical presentation between patients with oligoarticular JIA and LA and highlights the importance of Lyme antibody testing in all patients presenting with mono- or oligoarticular arthritis in endemic regions. With the continued rise in LA cases in Western New York, it is essential for providers across the state to be aware of the distinct clinical and laboratory features of Lyme arthritis and oligoarticular JIA and maintain a high suspicion for both in pediatric patients presenting with mono or oligoarticular joint arthritis.

## Conclusions

LA shares features with oligoarticular JIA. This overlap prevents the creation of a clinically useful predictive model for LA. Therefore, Lyme testing should be performed on all patients presenting with mono- and oligoarticular arthritis. In addition, ESR, CRP, and WBC in the synovial joint fluid were significantly higher in LA compared to oligoarticular JIA in our findings. While this difference is not definitive by any means, it may help distinguish between the two in cases where the diagnosis is not clear-cut, and the values of ESR, CRP, and WBC in the joint aspirate may help guide clinical judgment in cases that lack a definitive diagnosis.
